# Endothelial Transmigration by *Trypanosoma cruzi*

**DOI:** 10.1371/journal.pone.0081187

**Published:** 2013-12-02

**Authors:** Bria M. Coates, David P. Sullivan, Ming Y. Makanji, Nga Y. Du, Cheryl L. Olson, William A. Muller, David M. Engman, Conrad L. Epting

**Affiliations:** Departments of Pediatrics and Pathology, Northwestern University, Chicago, Illinois, United States of America; Albert Einstein College of Medicine, United States of America

## Abstract

Chagas heart disease, the leading cause of heart failure in Latin America, results from infection with the parasite *Trypanosoma cruzi*. Although *T. cruzi* disseminates intravascularly, how the parasite contends with the endothelial barrier to escape the bloodstream and infect tissues has not been described. Understanding the interaction between *T. cruzi* and the vascular endothelium, likely a key step in parasite dissemination, could inform future therapies to interrupt disease pathogenesis. We adapted systems useful in the study of leukocyte transmigration to investigate both the occurrence of parasite transmigration and its determinants *in vitro*. Here we provide the first evidence that *T. cruzi* can rapidly migrate across endothelial cells by a mechanism that is distinct from productive infection and does not disrupt monolayer integrity or alter permeability. Our results show that this process is facilitated by a known modulator of cellular infection and vascular permeability, bradykinin, and can be augmented by the chemokine CCL2. These represent novel findings in our understanding of parasite dissemination, and may help identify new therapeutic strategies to limit the dissemination of the parasite.

## Introduction

*Trypanosoma cruzi* is the etiologic agent of Chagas disease, a major disorder of poverty in the Americas that afflicts 10–12 million people [1]. There is a wide range of clinical sequelae, including lifelong asymptomatic infection, development of gastrointestinal pathology, acute fulminant myocarditis or encephalitis, and the development of chronic inflammatory cardiomyopathy [2], [3]. In the chronically infected individual a restricted tissue pool involving cardiac muscle and enteric nerves develop pathology [4]. The early diagnosis and treatment of Chagas disease remains a challenge for resource-poor nations, with the acute phase often passing undetected, and therapy during the chronic phase being largely supportive rather than curative [4]–[7]. Treatment options remain toxic and poorly efficacious, and no approved vaccines are presently available [8]–[10].

*T. cruzi* typically gains access to its human or animal host when an insect vector introduces infective metacyclic trypomastigotes into a wound or mucous membrane. After establishing a primary infection in the local tissues, the parasites spread via hematogenous dissemination [11]. Although the parasite is capable of infecting nearly any nucleated cell *in vitro*, in the chronically infected host, symptomatic pathology disproportionally impacts the heart and enteric nerves [7], [12]. Preferential binding of a *T. cruzi* surface protein motif on Tcgp85 to the lectin-binding galectin-3 on the cardiac vasculature may link endothelial homing to cardiac tropism [5]. Differential association with various organ beds offers an attractive hypothesis to explain apparent tissue tropism. Emerging literature addresses the vascular-parasite interface, including the role of the endothelin-1 and bradykinin pathways [13], [14]. Nerve growth factor receptors, specifically TrkA and C, have specifically been implicated in parasite persistence and cardiomyocyte infection, respectively [15], [16]. Recently, prostanoid-receptors on the parasite surface further implicated the thromboxane pathway during invasion [17]. The connections between *T. cruzi* infection, disease pathogenesis, and inflammatory pathways are well established [18]. As the initiation, propagation, and maintenance of the inflammatory response relies on the endothelial interface, further study of endothelial-pathogen interactions is warranted. Many inflammatory molecules are involved in the host response to infection, many cytokines, chemokines, lipid moieties, and modulators of nitric oxide [19], [20]. Interestingly, the chemokine CCL2 has been reported to increase *T. cruzi* recruitment *in vivo* [21], suggesting that pre-existing inflammation may help regulate subsequent tissue infection.

Dissemination of the parasite is a critical step in host infection and tissue targeting. While the process of cellular infection and replication has been well studied [20], [22], [23], virtually nothing is known about how *T. cruzi* crosses the endothelial barrier [24]. The endothelium freely allows the passage of water, ions, and small molecules, yet restricts the movement of proteins and prevents the passive transit of virtually all large macromolecules and cells (i.e. platelets and leukocytes) [25]–[27]. To circumvent this barrier, leukocytes have adapted specialized mechanisms that involve sequential interactions between adhesion molecules on both the leukocyte and endothelial cell. Leukocyte transmigration typically occurs at endothelial cell junctions and has been shown to require PECAM, CD99, and several other proteins [28]–[30]. Although acute inflammation is typically associated with localized increases in vascular permeability to fluid, regulated leukocyte transmigration proceeds without a profound alteration of barrier function, suggesting that transmigration is a tightly regulated process [26], [28]. Furthermore, even in inflamed tissue where vascular permeability is the highest, leukocyte transmigration is still dependent on PECAM and CD99, indicating that transmigration is an active process, not simply passive diffusion.

While trypomastigotes are significantly smaller and more motile than leukocytes, they are still several orders of magnitude larger than molecules able to passively diffuse across the endothelial barrier. As such, the endothelium represents a significant barrier for parasite entry into target tissues. Penetration of the endothelial barrier by pathogens is an emerging field, and various pathogens contend with the barrier using different strategies [31], [32]. We speculated that there were at least three active mechanisms by which *T. cruzi* could escape the blood stream. First, *T. cruzi* could cause physical disruption of the endothelial barrier, opening holes in the endothelium to permit direct parasite passage. While inflammation of the endothelium has been established during chronic infection [24], and certainly contributes to endothelial injury, microthrombi, and coronary ischemia, such a destructive process for initial passage would likely cause diffuse endothelial dysfunction, a disturbance of coagulation, and micro-hemorrhage. Such a gross disruption has not been described, and might be maladaptive for the establishment of a chronic infection. Alternatively, the parasite could serially infect and lyse cells, penetrating deeper with each release cycle. This is a highly inefficient process requiring many days, yet data suggests that host tissues are rapidly infected following dissemination [33], [34]. A third alternative would employ regulated transmigration across the endothelium, potentially co-opting pathways common to leukocyte transmigration, macromolecular nutrient uptake, or phagocytosis. Such transmigration would leave the endothelial barrier intact, and could occur between cells (paracellular at cell junctions) in a fashion similar to leukocyte transmigration, or through cells (transcellular) in a process that diverges from canonical cellular infection. Thus, we hypothesized that *T. cruzi* has the ability to cross the endothelium without disrupting the endothelial barrier, that this occurs in a matter of hours instead of days, and that this might be facilitated by pathways regulating cellular infection and endothelial permeability.

Changes in endothelial permeability can occur both across individual cells and at the junctions between cells. The *T. cruzi* virulence factor, cruzipain, is known to cleave human kininogen (HK) into bradykinin, an inflammatory mediator of endothelial permeability [35]–[37]. Although extravasation of fluid across the endothelium is seen with bradykinin challenge, whether this permeability change is sufficient to allow passage of the parasite is unknown. Additionally, the exact mechanism of bradykinin-induced permeability and the site at which it occurs remains unclear [38], [39], although caveolae are suspected to be involved. In this study, we first tested whether *T. cruzi* could bypass productive infection of endothelial cells (EC) and migrate across an endothelial monolayer. We then examined how cruzipain liberation of bradykinin might manipulate the endothelial monolayer to permit this process. We sought to identify the site of transmigration through evaluation of the transcellular and paracellular pathways. We then investigated the effect of the chemokine CCL2, a known chemoattractant for *T. cruzi* [21], on the kinetics of endothelial transmigration, to explore the potential role of inflammation in tissue tropism. Finally, we investigated parallel behavior in related Trypanosomatids to provide specificity of this process.

## Materials and Methods

### Ethics Statement

All animal-based experiments were specifically reviewed and approved by the animal care and use committee (ACUC protocol 2012-1009) at Northwestern University, carrying an IACUC assurance number A3283-01. Human subject research was reviewed and approved by the Institutional Review Board for Protection of Human Subjects at Northwestern University. Human tissues, from discarded placental samples, were collected in accordance with an IRB approved protocol involving consent-exempt research (Section 45 CFR Part 46.4). Northwestern University and Northwestern Memorial Hospital comply with Federal wide Assurance (FWA00001549, OMB 0990-0278) with the Office for Human Research Protections and the Federal Drug Administration in the Department of Health and Human Services.

### Reagents

Sources for reagents were as follows: Captopril (Acros Organics); HOE 140 (Tocris Bioscience); Recombinant human kininogen (high molecular weight), recombinant human CCL2 and E-64, (R&D Systems); FITC-dextran, sodium azide (10 mM), deoxyglucose (1 mM), thapsigargin, and wortmannin were all obtained from Sigma-Aldrich. Reagents were solubilized and stored according to manufacturer recommendations. Monoclonal mouse anti-human PECAM-1 (clone hec7) and monoclonal mouse anti-human CD99 (clone hec2) have been described previously [40], [41].

### Mammalian cell culture

Human umbilical vein endothelial cells (HUVEC) were isolated from de-identified fresh human umbilical cords as described [42] and grown in M199 medium (Invitrogen) supplemented with 20% heat-inactivated normal human serum and 100 U/mL penicillin–streptomycin at 37°C in a humidified atmosphere of 5% CO_2_. HUVECs at passage two were cultured at 2×10^4^ cells/well on thick hydrated collagen type I (Vitrogen; Cohesion Technologies) matrices set in 96-well plates [43]. Confluence and monolayer integrity of HUVECs on collagen matrices was assessed prior to use with Wright-Giemsa staining (Hema 3 kit, Fisher Scientific) and phase microscopy [43].

### Animals and parasites

Eight week-old male A/J mice (Jackson Laboratories, Bar Harbor, ME) were housed under specific pathogen-free conditions. Mice were infected by intraperitoneal injection of 1×10^4^ *T. cruzi* Brazil strain trypomastigotes derived from infected H9C2 rat myoblast cultures (American Type Culture Collection, Manassas, VA) that were maintained as described previously [44]. After 14 days of infection, mice were anesthetized by a single intraperitoneal injection of tribromoethanol (140 mg/kg) prior to euthanasia, the hearts removed, minced, and placed in co-culture with H9C2 cells. The following day, cultures were rinsed twice with PBS, and emerging trypomastigotes were collected on Day 5–6 for re-infection and expansion through H9C2. Culture-derived parasites were harvested from infected H9C2, centrifuged at 250 x *g* to remove cellular debris, then supernatants centrifuged at 3800 x *g* to pellet parasites. To maintain consistent infectivity, parasites were used until passage 5 (out from animal derivation) and then discarded, with HUVEC infections occurring between passages 3 and 5.

*T. brucei brucei* bloodstream form “single marker” strain [45] parasites were cultured at 37°C with 5% CO2 in HMI-9 medium [46], supplemented with 10% FBS, 10% serum plus medium complement (SAFC Biosciences), 100 U/mL penicillin/streptomycin, and 15 µg/mL G418. Cultures were periodically sub-cultured to maintain log-phase growth between 1×10^5^/mL and 2.5×10^6^/mL. Cells were counted and centrifuged at 1500 x *g* for buffer exchange into pre-warmed HUVEC media immediately prior to addition to the monolayers and 1×10^5^ parasites/sample were used for all assays.

*L. major* promastigotes [47] were grown to a density of 5×10^7^ cells/mL at 26°C in M199 media supplemented with 10% heat-inactivated FBS, then centrifuged at 1500 x *g* for buffer exchange into pre-warmed HUVEC media immediately prior to addition to the monolayers and 1×10^5^ parasites/sample in 100 µl were used for all assays.

### Quantification of transmigration and fluorescence microscopy

Trypomastigotes were resuspended in HUVEC growth media at 1×10^6^/mL. Where indicated, parasites and confluent endothelial monolayers were separately pre-treated with agonists or inhibitors at the specified concentrations in HUVEC media for 30 minutes. For the infection and transmigration studies, the media was removed from the endothelial cells and replaced with 100 µL of the indicated trypomastigotes. Samples were incubated at 37°C in the incubator for 2–3 hr. For treated samples, the reagents were present throughout the incubation. After the incubation, monolayers were washed with PBS and fixed in freshly prepared 2% glutaraldehyde (Sigma) overnight, washed again with PBS and quenched with 100 µM glycine (Sigma).

For scoring infection and transmigration, monolayers were then permeabilized with 90% methanol and stained with Wright-Giemsa (Hema 3 kit, Fisher Scientific) [43]. The collagen-supported monolayers were then removed from the wells and mounted on slides for visualization using bright field microscopy at 100x magnification. Both transmigrated parasites and those associated with the endothelial monolayer were manually counted. At least 150 parasites or 20 fields were counted for each endothelial monolayer. Three to six replicates were scored for each condition. Data were expressed as either the percentage of transmigrated parasites per high-powered field compared to the total number of parasites (i.e., those associated with the monolayer and those in the collagen matrix), or as the fold change in number of transmigrated parasites per high power field normalized to control.

For immunofluorescence imaging, monolayers were mounted in ProLong Gold antifade reagent with DAPI (Invitrogen). Images were acquired on a Zeiss Z1 AxioImager at 100x magnification under oil (Primo Star Plan-ACHRO objective, Zeiss) to identify parasites and host cells via phase microscopy and DAPI staining, and processed using Axiovision. Maximum intensity projections were reconstructed from a Z-stack of sequentially obtained 0.2 µm slices.

### TEM and CCL2

Collagen matrices supporting endothelial monolayers were pre-equilibrated in CCL2 at specified doses for 1 hour prior the addition of trypomastigotes. Human kininogen, E-64, and HOE 140 were added as indicated for the last 30 min. Monolayers were then rinsed twice with PBS, leaving behind a CCL2 gradient in the HUVECs and the collagen matrix. Parasites were then added with the indicated reagents and incubated and processed as described above.

### Permeability analysis

HUVEC grown on collagen matrices as described above, or collagen matrix alone, were incubated with FITC-conjugated dextran (78 MW, concentration) in the growth media with or without *T. cruzi* trypomastigotes for 3 hours. Unincorporated parasites and FITC-Dextran were removed with two washing in PBS, followed by fixation in 2% glutaraldehyde. Total retained FITC signal (cells + collagen matrix) was measured using a top-reader fluorescence plate reader (Gemini XS with SoftMaxPro software).

### Statistical analysis

Data were analyzed using Prism5 (Graph Pad) software’s unpaired t-test with Welch’s correction to account for any difference in variances between the samples. Data are expressed as mean ± SEM. *P*<0.05 was considered significant.

## Results

### *T. cruzi* undergoes transendothelial migration

**Figure 1:**
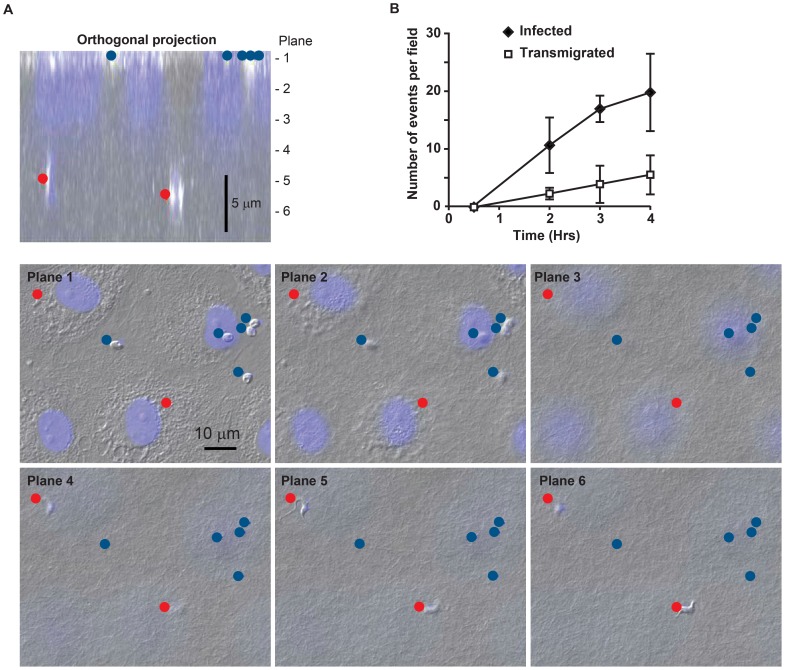
*T. cruzi* migrates across an endothelial monolayer. (A) Endothelial cells (EC) grown on three dimensional collagen matrices were fixed and stained with DAPI to visualize nuclei. Samples were imaged using phase contrast and immunofluorescence microscopy. A typical control sample is shown. Parasites that had infected the ECs (blue dot) were found in the same focal plane (Plane 1 in the image) as EC nuclei (shown in blue). Parasites that underwent transendothelial migration were identified based on their morphology within the collagen matrix up to ∼10 µm below the EC monolayer (red dot, Planes 4 and 6). A projection of the cross-section is shown for orientation. Typically, H&E was used to score, DAPI stained sections with phase are shown here for clarity. (B) To monitor the time course of infection and transmigration separate plates of control samples were incubated for the indicated times before the reaction was stopped by fixation and quantified as described in the materials and methods. Data shown were collected from three experiments with multiple replicates per experiment.

To investigate the ability of *T. cruzi* to transmigrate across an endothelial monolayer, we adapted a standard *in vitro* system used to study leukocyte transmigration. In this approach, primary endothelial cells (EC) are grown on top of a collagen matrix in a 96-well plate [43]. These endothelial cells exhibit contact-inhibited growth, recapitulate appropriate barrier function, and express all of the standard markers (i.e. ICAM-1, PECAM, cadherin-5) making this model an accurate representation of a true endothelium [40], [43], [48]. Furthermore, all factors and treatments known to affect endothelial permeability and leukocyte transmigration *in vivo* have similar effects in this *in vitro* model, suggesting that this experimental setup properly mimics a vascular bed. To assay *T. cruzi* transmigration, confluent monolayers were incubated with parasites for 2-3 hours, after which the samples were washed rapidly, fixed and stained. Transmigration and infection were scored by staining samples with hematoxylin and eosin (H&E) or with DAPI for phase and fluorescent microscopy, thus enabling determination of parasite depth relative to the monolayer. As expected, *T. cruzi* readily infected EC and were easily identified in the plane of the monolayer (Figure 1A). The majority of these infecting parasites had the typical early amastigote morphology characteristic of the vacuole-encased parasite. Interestingly, over the course of the incubation approximately 10–30% of the parasites were observed with trypomastigote morphology in planes well below the EC monolayer, an event henceforth referred to as transendothelial migration (TEM). A movie demonstrating the depth of penetration of the parasite into the collagen matrix is presented as supplemental material (Movie S1).

The benefit of this *in vitro* model of parasite TEM is that it is a robust way to examine the interaction of *T. cruzi* with a major barrier encountered during parasites dissemination. To our knowledge this is the first report of such a model in the study of *T. cruzi*, thus it was necessary to characterize the system. Although TEM was infrequent after short incubation times (i.e. 5–30 minutes), by 2 hours both infection and TEM were apparent and increased in frequency at a linear rate out to 4 hours, the latest time point analyzed (Figure 1B). Studies showed that infection and transmigration remained proportional to each other over a range of parasite concentrations (MOI 1:10–1:30) and incubation times. For consistency, the experiments reported here were performed with 1×10^5^ parasites in 100 µl per well involving a 2–3 hour infection. This resulted in a consistently measurable number of TEM events over a short time period, with 30% or more of the parasites either infecting the EC or undergoing TEM.

### *T. cruzi* infection does not alter EC monolayer permeability

**Figure 2:**
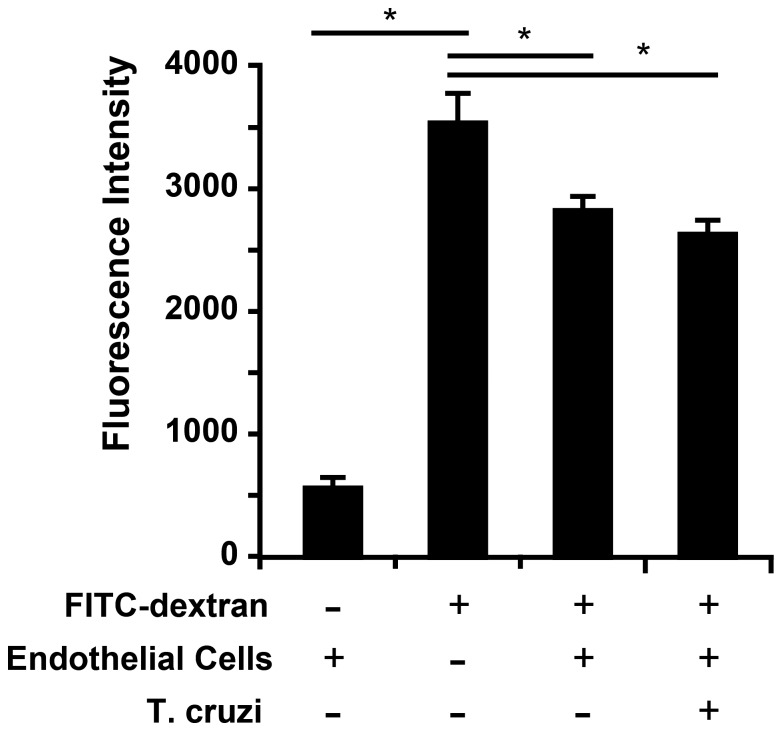
Permeability is not altered during *T. cruzi* infection and TEM. EC monolayers were incubated with *T. cruzi* identical to the standard TEM reaction except that 100 µg/mL FITC-dextran was included in the media to assay monolayer permeability. After a three hour incubation, samples were washed extensively and the amount of FITC-dextran that had crossed into the collagen matrix was quantified using a fluorescence spectrophotometer. Data shown represent the mean and standard deviation of six individual replicates. * denotes p<0.05 for the indicated comparisons.

Because the *T. cruzi* surface is rich in proteases, we reasoned that parasites could be crossing the endothelium following a disruptive event damaging either the monolayer or individual EC junctions. Immunofluorescence microscopy of HUVEC monolayers following infection did not reveal any gross disruption of the EC or overt retraction of the junctions following the addition of parasites. This suggested that *T. cruzi* did not undergo TEM by simply destroying the monolayer. *T. cruzi* TEM could operate by a mechanism that is similar to leukocyte TEM, during which neutrophils and monocytes move discreetly between cells via gaps induced at EC junctions. To examine if *T. cruzi* causes similar gaps during TEM, we stained samples after incubation with *T. cruzi* with antibodies against PECAM, a marker of endothelial junctions, and examined junctional integrity by immunofluorescence microscopy. No difference was observed between monolayers incubated with *T. cruzi* and controls (data not shown); both samples showed contiguous uninterrupted PECAM staining at all endothelial junctions. To exclude the possibility that transient gaps were created, yet closed by the end of the experiment, we conducted the TEM assay in the presence of fluorescently conjugated FITC-dextran. Thus, any significant changes in the monolayer integrity would be observed by leakage of labeled dextran into the collage gel. After fixing and washing the samples, the amount of dextran that had leaked through the monolayer was quantified using a fluorescence plate reader (Figure 2). Consistent with other reports, ECs alone were relatively permeable to labeled dextran, but formed a significant barrier when compared to collagen without an EC monolayer. Addition of parasites for up to three hours had no significant effect on the permeability of the monolayer, despite the monolayer having endured an estimated 10,000 TEM events (calculated using the measured TEM rate and monolayer size in parallel samples). Similar experiments were conducted measuring the transendothelial voltage in response to parasite infection in a 96-well transwell apparatus (EndOhm, World Precision Instruments). No significant change in voltage was detected during the first two hours of infection, which paralleled the results obtained using FITC-dextran (data not shown). *T. cruzi* disrupts EC junctions during TEM, then it must be done in a carefully controlled, easily repaired manner that does not result in the either the destruction of the monolayer or static separation of the junctions. Indeed the gaps induced during leukocyte TEM are rapidly closed behind the migrating cell, and do not generally result in a change in permeability [49].

### Parasite TEM differs from leukocyte transmigration

**Figure 3:**
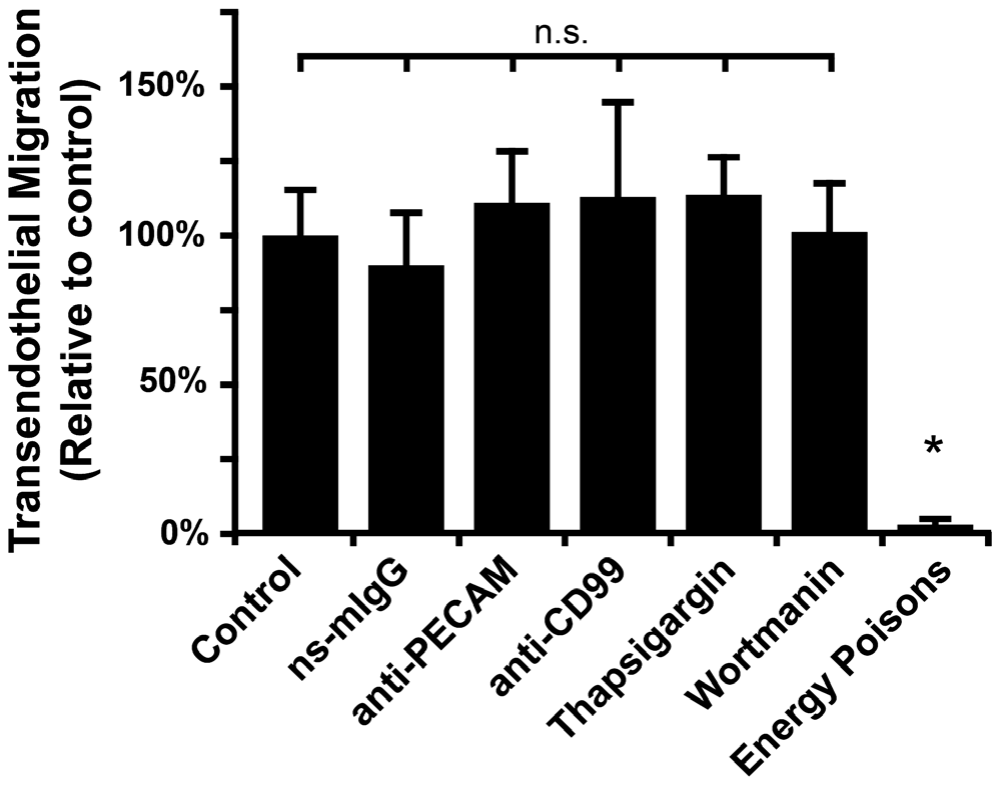
Elucidating determinants for *T. cruzi* TEM. ECs and *T. cruzi* were pre-incubated with the indicated reagents for 30 min prior to infection. Non-specific mouse IgG (ns-mIgG), mouse anti-human PECAM, and mouse anti-human CD99 antibodies were used at 20 µg/mL. Thapsigargin and wortmannin were used at 10 mM and 100 nM, respectively. To disrupt cellular energy production, samples were treated with 10 mM sodium azide and 6 mM deoxyglucose. After adding the parasites to the ECs, samples were then incubated for 3 hours in the continuous presence of the reagents before being washed, fixed and scored for transmigration as described. Data shown are the mean and standard error of the mean for three separate experiments with 2–3 replicates for each condition per experiment. * denotes p<0.05 relative to the control.

We next sought to identify potential mechanisms of this non-destructive migration of *T. cruzi* across the endothelium. Leukocytes also migrate across the endothelium without cellular destruction or disruption, so we first sought to determine if *T. cruzi* has co-opted the leukocyte TEM pathways. PECAM and CD99 are required for efficient leukocyte TEM in humans [40]. Although both proteins are expressed at endothelial junctions where the majority of TEM occurs (termed paracellular TEM), they are also required for TEM that occurs through the endothelial cell body (termed transcellular TEM) [50], [51]. To determine if *T. cruzi* TEM engages the same molecules, we assayed parasite TEM in the presence of function-blocking antibodies against PECAM and CD99. These treatments have been shown to disrupt >80% of leukocyte TEM [40], [48], [51], however, they had no effect on *T. cruzi* migration across the endothelium (Figure 3) or infection of the ECs (data not shown). These data suggest that *T. cruzi* are able to cross the endothelium by a mechanism that is distinct from leukocyte TEM and does not involve PECAM and CD99.

### Identifying determinants of parasite TEM

Having shown that *T. cruzi* undergoes TEM by a pathway that is distinct from leukocytes, we examined other possible pathways that the parasite could exploit to cross the endothelium. Non-productive infection, where the parasite enters a cell but avoids the canonical infection pathway that leads to differentiation and replication, has been described [52]. In these cases, after entering the cell, the nascent parasitophorous vacuole that surrounds the trypomastigote fails to fuse with lysosomes, the motile parasite cannot be retained in the cell, and it is seen to exit prior to differentiation and replication. We hypothesized that *T. cruzi* passes through the endothelial cell by way of a non-productive infection, with its exit occurring on the basal-lateral rather than apical surface of the endothelial cell, thus gaining access to the matrix (or tissue) below the monolayer. Such a pathway should respond to published infection inhibitors, with TEM being downstream of cellular invasion, thus agents that modify initial interaction or invasion should simultaneously impact infection and transmigration. We examined TEM in samples incubated in the presence thapsigargin and wortmannin, which disrupt endoplasmic reticulum (ER) calcium release and phosphoinositide 3- kinases (PI3K) signaling, respectively. These agents have both been established to impair infection [53], [54]. Neither treatment had a significant effect on the ability of *T. cruzi* to breach the EC monolayer (Figure 3), suggesting that TEM was determined upstream of the PI3K or ER calcium pathway. This result led us to suspect that TEM could be a passive process, however, incubating samples with the combined energy poisons sodium azide and deoxyglucose during TEM to deplete EC and *T. cruzi* energy and ATP stores almost completely abolished both TEM and infection (Figure 3 and data not shown). Additionally, heat inactivated *T. cruzi* did not undergo TEM (data not shown). Taken together, these results suggest that *T. cruzi* TEM requires the active energetics of both the host and the parasite, and that TEM determinants are upstream of thapsigargin or wortmannin sensitive steps in infection.

### Bradykinin signaling contributes to parasite TEM

**Figure 4:**
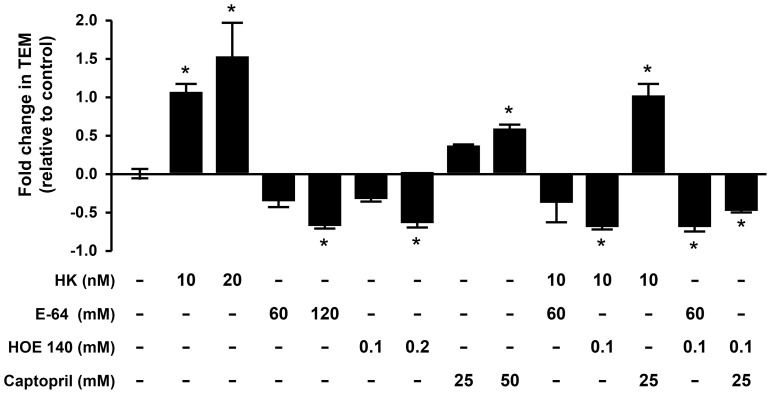
Bradykinin augments *T. cruzi* TEM. Agonists and inhibitors of the bradykinin pathway were pre-incubated with parasites and EC for 30 min at the indicated concentrations before combining the two for the standard TEM assay. TEM was allowed to proceed for 3 hours in continuous presence of the reagents. Samples were then washed, fixed and scored as described in the Methods. Data were collected from three experiments with 2–3 replicates per experiment. To correct for variations between parasite preparations and aid in visualization, data are shown as the fold change relative to control (no treatment). * denotes p<0.05 relative to the control.

Our next approach to identify the pathways involved with parasite TEM was to broaden our search and examine factors thought to be upstream, thus as those involved in the initial cell-cell interaction or *in vivo* dissemination. The *T. cruzi* virulence factor cruzipain, a cysteine protease, increases parasite infectivity through cleavage of human kininogen (HK) into bradykinin, and the activation of the bradykinin receptor 2 (B_2_R) pathway [35]–[37]. To test whether this pathway was involved in TEM, we added agonists and antagonists of the B_2_R pathway to our EC model of TEM. Indeed, incubating samples with the substrate HK significantly increased the amount of parasites that crossed the EC monolayer relative to control (Figure 4). To account for infection variability observed between experiments performed on different days, the data from each experiment were normalized to the corresponding internal control, no treatment. The average fold-change relative to the internal controls is shown. To confirm that TEM was being modulated by bradykinin signaling, we treated EC with the B_2_R antagonist HOE 140 [37]. Pharmacologic inhibition of the bradykinin receptor significantly reduced the ability of parasites to cross the endothelium indicating that signaling through this receptor is required for efficient TEM. Likewise, inhibiting cruzipain activity with E-64, a cysteine protease inhibitor and known inhibitor of cruzipain, which reduces the conversion of HK into bradykinin [37], also significantly decreased TEM. Further supporting the involvement of this pathway is our observation that treatment with captopril, an angiotensin-converting enzyme (ACE) inhibitor that inhibits the breakdown of bradykinin [35], [37], thus prolonging its effects, increased TEM.

Consistent with these findings are the observations that HK alone was incapable of augmenting TEM when the bradykinin receptor was inhibited by HOE 140, or when cruzipain-mediated cleavage of HK into bradykinin was inhibited with E-64 (Figure 4). Similarly, preventing bradykinin degradation with captopril was ineffective when bradykinin receptor activity was blocked with HOE 140 or when bradykinin production was inhibited. Treatment with both HK and captopril did not augment transmigration further indicating that the maximal TEM effect was achieved with either alone. Similarly, combining both HOE 140 and E-64 did not further inhibit TEM, supporting the shared pathway for these inhibitors, and that the maximal effect could be achieved with either alone. Combined, these data indicate that *T. cruzi* cysteine protease-dependent cleavage of HK into bradykinin, and signaling through B_2_R, facilitates parasite TEM.

### The chemokine CCL2 promotes TEM

**Figure 5:**
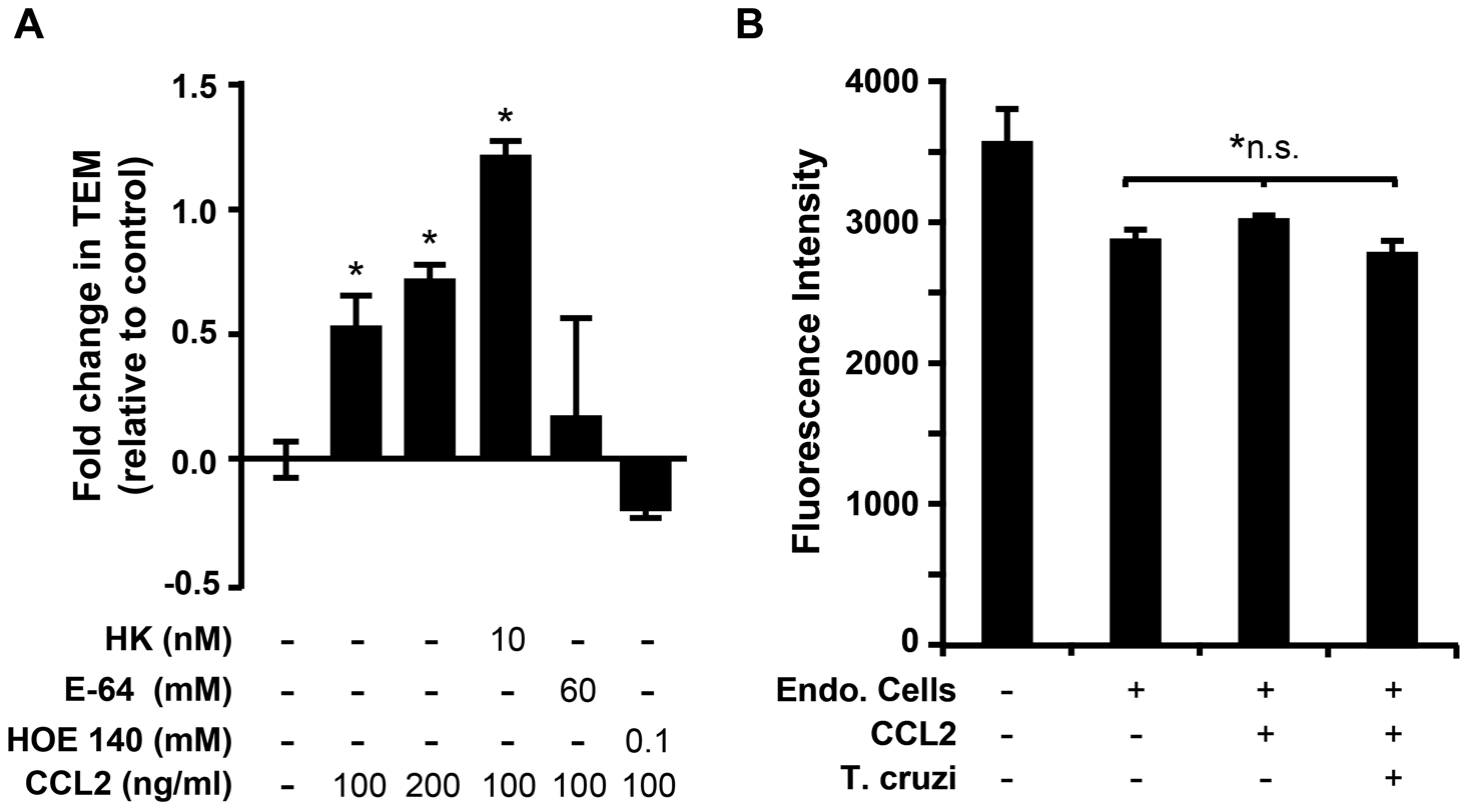
CCL2 augments *T. cruzi* TEM. (A) To assess the effect of the chemokine CCL2 on TEM, EC monolayers were pre-incubated with CCL2 for 1 hour before the start of the standard TEM assay. Samples were washed briefly before the start of TEM, leaving a gradient of CCL2 in the collagen matrix. Other reagents were also pre-incubated with parasites and EC (in addition to CCL2) for 30 min. at the indicated concentrations before the start of the assay. After adding the parasites to the monolayers, TEM was allowed to proceed for 3 hours. Samples were incubated in the continuous presence of the reagents, except CCL2, which was only present during pre-incubation. Samples were then washed, fixed and scored as described in the Methods. Data were collected from three experiments with 2–3 replicates per experiment. To correct for variations between parasite preparations and aid in visualization, data are shown as the fold change relative to control. * denotes p<0.05 relative to the control. (B) Permeability was quantified in samples that were identical to those in panel (A) except that 100 µg/mL FITC-dextran was included in the media during the incubation. After washing and fixation, the amount of FITC-dextran that had crossed the monolayer into the collagen matrix was quantified using fluorescence spectrophotometry. Data represent the average and standard deviation of six independent replicates. *n.s. - None of the indicated samples were statistically significant (p <0.05) relative to each other, and CCL2 treatment (200 nM) under these conditions did not significantly alter monolayer permeability in the presence or absence of *T. cruzi* (Figure 6B compared to Figure 2).

The chemokine CCL2 has been reported as a chemoattractant for *T. cruzi in vivo*, where the addition of CCL2 to a sterile air pouch significantly increased the number of parasites recovered at the site following lavage [21]. Chemokines and other chemoattractants are well studied by similar endothelial monolayer systems in leukocyte biology. We hypothesized that CCL2 should augment parasite TEM in our simplified *in vitro* system. Toward this end, we established a CCL2 gradient by pre-incubating the ECs and collagen with CCL2, which, after washing, leaves a residual gradient across the monolayer. Indeed, the presence of CCL2 considerably increased *T. cruzi* TEM in a dose dependent manner (Figure 5A). This effect was additive with HK suggesting that the CCL2 mechanism likely operates by a pathway distinct from the bradykinin-mediated pathway. However, CCL2 augmentation of TEM was abolished by blocking HK cleavage into bradykinin (by E-64) and by inhibiting bradykinin receptor. This observation cannot be explained simply by the effect of CCL2 on permeability as the conditions used in this assay had no change on the permeability of FITC-dextran with or without CCL2 or parasites (Figure 5B).

### Specificity of TEM for related trypanosomatids

**Figure 6:**
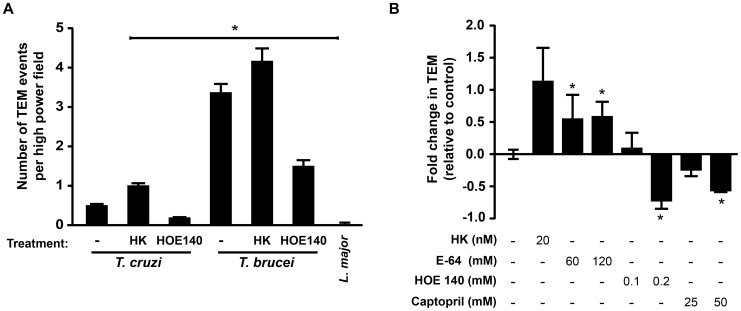
Comparative TEM among the *Kinetoplastidae*. (A) TEM was assayed for *T. cruzi*, *T. brucei*, and *Leishmania major* using our standard TEM method with 1×10^5^ parasites/well. Parasites were prepared as described in the Methods. Where indicated, samples were treated (both during a 30 minute pre-incubation and during infection/TEM) with HK or HOE 140 at 20 ng/mL and 200 nM, respectively. Data shown represent the total TEM events per high power field to highlight the large differences observed with *T. brucei* and *L. major*. Data were collected from three independent experiments with 2–3 replicates per sample per experiment. * denotes significant difference relative to *T. cruzi* non-treated controls, p <0.05. (B) To confirm that *T. brucei* TEM was responsive to manipulations of the bradykinin signaling pathway, we treated samples with the indicated reagents (both pre-incubation and during TEM) and assayed TEM using the standard assay**.** To correct for variations between parasite preparations and aid in visualization, data are shown as the fold change relative to control (no treatment). * denotes p<0.05 relative to the control.

To investigate the specificity of TEM to *T. cruzi*, transmigration was assessed with related pathogens, *Leishmania major* and *Trypanosoma brucei*. Although virtually no TEM was observed with *L. major*, *T. brucei*, which has been shown to cross the blood-brain barrier [55], rapidly transmigrated across EC (Figure 6A). *T. brucei* express the protease brucipain (cathepsin L), a homolog of cruzipain, the latter of which has also been shown to liberate bradykinin from kininogen [55]. We reasoned the *T. brucei* TEM could also be modulated by reagents that affect bradykinin signaling. As expected, *T. brucei* TEM was affected by HK, E-64 and HOE 140 in a manner that mirrors that of *T. cruzi* (Figure 6B, compare to Figure 4), suggesting that these two parasites likely employ a similar mechanism to cross the endothelium.

## Discussion

Although *T. cruzi* disseminates intravascularly, little is known about how the parasite breaches the endothelial barrier. Delineation of this process could identify new therapeutic targets to limit the ongoing infection into targeted tissues. In an effort to investigate how *T. cruzi* escapes the vasculature, we evaluated the interaction of *T. cruzi* with an endothelial monolayer in a well-established *in vitro* model of leukocyte TEM. Our model does not include flow conditions, but is widely accepted as a good representation of the biology of post-capillary venules [40], [43], [48]. Here we show that *T. cruzi* can, in fact, bypass canonical infection pathways that lead to parasite differentiation and replication within host cells, and instead pass through what is normally an extremely effective cellular barrier to macromolecule transit. Importantly, the transendothelial migration of *T. cruzi* is not associated with overt disruption of the endothelial monolayer, and is not associated with a change in endothelial permeability. However, even though *T. cruzi* TEM does not occur through destruction of the endothelium, it is an active process that cannot occur in the presence of energy poisons or heat-inactivated parasite.

Technical limitations prevent a thorough analysis into the specific route(s) of *T. cruzi* TEM. The depth of the collagen matrix necessary for this TEM model impairs live microscopy, but is indispensable for distinguishing between monolayer infection and the TEM event. The relative infrequency of events, the high magnification required, thus limiting the field and depth of view, and the necessity to witness the parasites exiting the cell, rather then entering, all contribute to the challenge of imaging these events. Without the ability to visualize the process, we instead defined it pharmacologically. Possible routes considered included paracellular (between cells) or transcellular (through cells). Coordinated paracellular transit in a manner akin to leukocyte transmigration is unlikely given the lack of effect of PECAM and CD99 blockade on *T. cruzi* TEM. Uncoordinated transit through paracellular junctions would be very difficult without disruption of the monolayer, although the involvement of a novel pathway cannot be excluded. Transcellular transit likely necessitates rapid intracellular movement of trypomastigotes without the complete formation of the parasitophorous vacuole, engagement of the host cytoskeleton, differentiation into amastigotes, and establishment of stable intracellular infection.

Parasite entry into host cells relies on molecular interactions at the membrane-membrane interface, utilizing a variety of pathways, which differ based on cell type (i.e. professional phagocytes) and parasite lifecycle stage (metacyclic trypomastigotes, cell-derived trypomastigotes, or amastigotes) [11], [56]. Emerging data reveals that parasite entry may trigger a membrane-repair process, in synaptotagmin VII-dependent process triggering the recruitment of lysosomes [57], via calcium-sensitive, PI3K-dependent pathways [58], [59]. Lysosomal fusion is a final common pathway leading to terminal cell infection [54], [60]. An alternative pathway resulting from cell entry without lysosomal fusion has also been described [52], [54]. Here the developing parasitophorous vacuole fails to fuse with lysosomes, and the trypomastigotes escape the cell without formation of the vacuole and differentiation into replicative amastigotes. To our knowledge, non-productive invasion has not been studied in a polarized cell monolayer, such as the endothelium, where an egress event could lead to apparent transmigration. This process would enable rapid “directional” dissemination of the parasite through the endothelial barrier. Our experiments do support that infection of the HUVEC was the dominant outcome, with 85–90% of events scored as intracellular parasites. Two mechanisms known to decrease *T. cruzi* infection of host cells, namely inhibition of PI3K and interruption of ER calcium release [53], [54], [59], did not prevent TEM, further suggesting that canonical pathways leading to lysosomal fusion are bypassed. It appears that TEM occurs via direct cell entry without lysosomal fusion and subsequent transcellular passage results from basolateral egress of the parasite. Definitive studies dissecting the downstream molecular events underlying TEM are clearly needed.

We found a striking dependence of *T. cruzi* TEM on bradykinin signaling involving kininogen, cruzipain, and the bradykinin-2 receptor. Substantial evidence connects kinin signaling to cell infection and Chagas vasculopathy [13], [61], although this has not been well explored in cultured primary endothelial cells. We initially postulated that bradykinin would increase solute permeability across the endothelium, although our studies did not reveal a significant change in permeability or electrical conductance during infection. Bradykinin is already known not to induce overt gap formation between cells [62], similar to our observation following parasite infection. The proposed mechanism of the bradykinin-induced increase in endothelial permeability is increased transcellular passage of solute [62], [63] through a caveolar-dependent pathway [64]. As bradykinin signaling has also been well studied to augment cell invasion, this supports our speculation that direct cellular invasion (transcellular passage) is upstream of TEM across the polarized monolayer [35]–[37]. The dependence upon caveolae for intracellular organisms has been well established [65], and *T. cruzi* regulates the expression of various caveolins proteins during infection. Mice deficient in caveolin-1/3 develop a cardiomyopathy reminiscent of Chagas heart disease [66], and evidence supports that the down regulation of caveolin may support cellular infection [67]. Given the links between caveolin, endothelial transport, and infection, a detailed examination of caveolar-dependent pathways during endothelial cell infection and transmigration is indicated.

Our finding that CCL2 augments TEM supports a role for this chemokine as a *T. cruzi* attractant [21]. The increase in TEM was dependent upon B_2_R, suggesting that bradykinin-mediated permeability enhances *T. cruzi* TEM towards a CCL2 gradient. Interestingly, *T. cruzi* harbors molecular mimics of cardiac myosin, which following peripheral inoculation initiate an autoimmune inflammatory process in the heart prior to parasite dissemination [68], [69]. Tissue specific inflammation might alter local EC permeability and liberate CCL2, thereby increasing *T. cruzi* recruitment and transmigration into the heart, potentially contributing to apparent tissue tropism. Cardiac tissues themselves are rich sources for inflammatory mediators during infection, and early events likely contribute to ongoing parasite invasion and persistence.

*T. cruzi* TEM likely contributes to the hematogenous dissemination into various tissues. It is facilitated by bradykinin, a modulator of vascular permeability, and is augmented by a chemoattractant. Bradykinin dependent TEM was also seen with *T. brucei*, but not *L. major*. This species specificity further supports our assertion that TEM is a specialized mechanism by which parasites can circumvent critical barriers to establish tissue infection. Part of the virulence conferred by cruzipain and its homologs may be related to its role in promoting efficient and rapid parasite dissemination. Further elucidation of the pathways involved in TEM will enhance our understanding of the biology of the parasite-host interface and potentially reveal novel therapeutic approaches. If the determinants of parasite TEM could be understood, and the mechanisms of tissue specificity elucidated, advancements in the prevention and treatment of a historically neglected disease may be achieved.

## Supporting Information

Movie S1 **Demonstrates serial phase/DAPI images taken at 0.5** µ**m increments starting at the top of the endothelial monolayer and penetrating through the cellular layer and into the collagen matrix, which appears as many fine fibers.** Note the encapsulated parasites in early vacuoles within the plane of the cell layer, and parasites in the trypomastigote form deeper in the collagen matrix.
(MOV)
